# Navigating Post-operative Challenges: A Comprehensive Review of Complications Following Anterior Cruciate Ligament (ACL) Tear Surgery

**DOI:** 10.7759/cureus.67768

**Published:** 2024-08-25

**Authors:** Milind R Gharpinde, Aditya Pundkar, Yash Dhanwani, Rohan Chandanwale, Ankit M Jaiswal

**Affiliations:** 1 Orthopaedics, Jawaharlal Nehru Medical College, Datta Meghe Institute of Higher Education & Research, Wardha, IND; 2 Orthopaedics, Jawaharlal Nehru Medical College, Datta Meghe Institute of Higher Education & Research, Akola, IND

**Keywords:** rehabilitation protocols, surgical outcomes, knee stability, graft failure, post-operative complications, acl reconstruction

## Abstract

Anterior cruciate ligament (ACL) injuries are among the most common and debilitating sports-related injuries, often necessitating surgical intervention to restore knee stability and function. ACL reconstruction surgery, which has evolved significantly over the years, aims to enable patients, particularly those who are young and physically active, to return to their pre-injury activity levels. However, despite advancements in surgical techniques and rehabilitation protocols, post-operative complications remain a significant concern that can adversely affect patient outcomes. This comprehensive review explores the spectrum of complications that can arise following ACL tear surgery, ranging from common issues such as infection, graft failure, and knee stiffness to less frequent but clinically significant complications like osteoarthritis and neurological injuries. The review also delves into the various factors influencing the likelihood of these complications, including patient-related variables, surgical techniques, and the effectiveness of rehabilitation protocols. By providing an in-depth analysis of these post-operative challenges, this review aims to enhance the understanding of ACL reconstruction outcomes and guide healthcare professionals in implementing preventive strategies and optimizing patient care. Through a multidisciplinary approach, the goal is to minimize the risk of complications, improve surgical outcomes, and ultimately enhance the quality of life for patients undergoing ACL reconstruction.

## Introduction and background

The anterior cruciate ligament (ACL) is one of the vital stabilizing structures of the knee joint, playing a crucial role in maintaining the knee's rotational stability and preventing anterior displacement of the tibia relative to the femur [1]. ACL injuries, particularly tears, are among the most common sports-related injuries, with an incidence rate that has increased over recent years due to the growing participation in high-impact sports [2]. These injuries often result from sudden stops, pivots, or changes in direction, making them prevalent among athletes involved in sports such as soccer, basketball, and skiing [2].

The impact of an ACL tear extends beyond the immediate injury, as it significantly compromises knee stability, leading to functional limitations, recurrent episodes of giving way, and an increased risk of secondary injuries, including meniscal tears and cartilage damage [3]. Without appropriate treatment, ACL injuries can contribute to the early onset of knee osteoarthritis, particularly in younger, active populations. The significance of ACL injuries lies not only in their frequency but also in their potential to cause long-term disability and hinder an individual's quality of life, particularly in those who are physically active [3]. Surgical intervention is often recommended for individuals with ACL tears, especially for those who are young, active or wish to return to sports that require knee stability. The primary goal of ACL reconstruction surgery is to restore the functional stability of the knee, thereby enabling patients to return to their pre-injury levels of activity while minimizing the risk of further injury. Surgery typically involves the reconstruction of the torn ligament using a graft, which can be harvested from the patient (autograft), a donor (allograft), or, less commonly, synthetic materials [4].

ACL reconstruction has evolved over the decades, with advancements in surgical techniques, graft choices, and postoperative rehabilitation protocols leading to improved patient outcomes. Despite these advancements, the decision to undergo surgery is multifactorial, considering the patient's age, activity level, presence of concomitant injuries, and personal goals. While many patients achieve successful outcomes following surgery, it is not without risks, and understanding the potential complications is crucial for informed decision-making and effective patient management [5]. The primary objective of this comprehensive review is to provide an in-depth analysis of the post-operative complications associated with ACL tear surgery. While the success rates of ACL reconstruction have improved, complications can still arise, impacting the overall outcome and quality of life of the patient. This review aims to explore the various complications that may occur following ACL surgery, ranging from common issues such as infection and graft failure to less frequent but significant problems like osteoarthritis and neurological injuries.

## Review

ACL tear surgery: overview

ACL reconstruction surgery is a prevalent procedure designed to restore stability and functionality to the knee following an ACL tear. The surgery typically involves replacing the torn ligament with a graft, which can be obtained from the patient’s own body (autograft), from a donor (allograft), or, though less commonly, from synthetic materials [6]. Commonly used autografts include the patellar tendon, hamstring tendon, and quadriceps tendon. The patellar tendon autograft involves harvesting a tendon segment and a small bone plug from the kneecap and shinbone. The hamstring tendon autograft uses tendons from the back of the thigh, while the quadriceps tendon autograft involves a portion of the tendon above the kneecap. Allografts, sourced from deceased donors, eliminate the need for additional surgical sites but may carry a higher risk of graft failure. Synthetic grafts are now used less frequently due to their higher failure rates [7]. Before ACL reconstruction, several pre-operative considerations must be addressed. Patient selection is critical and typically hinges on factors such as age, activity level, and the presence of other knee injuries. Younger and more active individuals often make better candidates for surgery, as they are likely to benefit more from restored knee stability [8]. Concurrent injuries, such as meniscus tears or cartilage damage, may also require surgical intervention. It is important for patients to understand that while ACL reconstruction can significantly enhance knee stability and function, it does not guarantee a complete cure. The primary goal of the surgery is to facilitate a safe return to desired activities and prevent further injury [8].

The expected outcomes following ACL reconstruction are generally favorable, especially with appropriate surgical techniques and diligent rehabilitation. Most patients can expect to regain full range of motion within two to three weeks post-surgery and may be able to discontinue crutches and the knee brace within two to four weeks [9]. Light sports activities can often be resumed within four to six months, with a return to full sports typically achievable within six to nine months. However, complications may arise, including graft failure rates of approximately 10-15%, and up to 30% of patients may not return to their pre-injury level of sports. Additionally, individuals who have undergone ACL reconstruction face an increased risk of developing osteoarthritis later in life [8]. Rehabilitation is crucial for optimizing outcomes and minimizing complications after ACL surgery. Physical therapy usually begins within the first week post-operatively, focusing on restoring motion, strength, and neuromuscular control. A gradual, sport-specific progression is essential to return to athletic activities safely. By adhering to a structured rehabilitation program and closely monitoring recovery, patients can maximize their chances of a successful outcome following ACL reconstruction surgery [10].

Common post-operative complications

Infection

Infection is a notable concern following ACL reconstruction, with incidence rates ranging from 0.1% to 2.4%. Several factors can increase the risk of infection, including male sex, obesity, tobacco use, diabetes mellitus, and a history of steroid use [11]. Additionally, prior knee surgeries and the type of graft used can affect infection rates, with hamstring autografts generally showing greater susceptibility than bone-patellar tendon-bone grafts. Clinically, infections commonly present with symptoms such as swelling, pain, reduced range of motion, drainage, and fever [12]. Diagnosis typically involves laboratory tests such as arthrocentesis, erythrocyte sedimentation rate, and C-reactive protein levels. Management strategies emphasize early detection and treatment, including conservative measures like oral antibiotics or more invasive procedures such as surgical debridement for severe cases. Preventive measures, including optimizing surgical techniques and postoperative care, are essential for reducing the risk of infection [12].

Graft Failure

Graft failure is a significant complication following ACL reconstruction, and it is classified into early and late failure. Early graft failure occurs within the first year after surgery and is frequently associated with surgical technique or improper graft placement. In contrast, late graft failure, which can happen after the first year, may result from biological factors such as insufficient healing or revascularization or mechanical issues like incorrect graft tension or alignment. When graft failure occurs, revision surgery may be required [13]. The outcomes of revision surgery can be favorable but are influenced by the underlying cause of the failure and the time elapsed since the initial procedure. Understanding the differences between early and late graft failures is crucial for developing effective management strategies and improving patient outcomes [14].

Knee Stiffness and Arthrofibrosis

Knee stiffness and arthrofibrosis are significant challenges in postoperative recovery, primarily resulting from excessive scar tissue formation. These conditions can be exacerbated by factors such as prolonged immobilization, inadequate rehabilitation, or surgical trauma. The pathophysiology involves a complex interaction of inflammation and fibrosis, which leads to a restricted range of motion [15]. Clinical assessment of knee stiffness typically includes a physical examination to evaluate the range of motion, and imaging studies may be employed to assess joint structure. Treatment options usually start with physiotherapy to restore motion; however, surgical intervention may be required to remove scar tissue and improve function in severe cases. Early intervention and a tailored rehabilitation program prevent stiffness and enhance recovery [16].

Deep Vein Thrombosis

Deep vein thrombosis (DVT) is a potential complication following ACL surgery, with risk factors including prolonged immobility, obesity, and a history of venous thromboembolism. The incidence of asymptomatic DVT after ACL surgery can be as high as 15% [17]. Clinically, DVT may present with swelling, pain, and tenderness in the affected leg, and diagnosis is typically confirmed through ultrasound imaging. Preventive measures are crucial in reducing the risk of DVT and include early mobilization, the use of compression stockings, and, when indicated anticoagulant therapy. If DVT occurs, treatment generally involves anticoagulation to prevent further complications, such as pulmonary embolism [18].* *Neurological complications, although relatively uncommon, can occur during ACL reconstruction surgery. The saphenous nerve is the most frequently affected, with incidence rates varying among studies. These nerve injuries can result in sensory deficits or motor weakness, significantly affecting rehabilitation and overall recovery [19]. Management of neurological complications typically involves physical therapy to enhance function, and, in some cases, surgical intervention may be necessary for nerve repair. Early recognition and appropriate rehabilitation strategies are crucial for optimizing recovery and minimizing the long-term impact of these complications. Table 1 outlines common postoperative complications following ACL tear surgery [20].

**Table 1:** Common Post-Operative Complications Following Anterior Cruciate Ligament (ACL) Tear Surgery

Complication	Description	Incidence Rate	Risk Factors	Management Strategies
Infection [21]	Bacterial contamination leads to localized or systemic infection.	0.5% - 2%	Poor hygiene, prolonged surgery, immunocompromised status	Antibiotics, surgical debridement, wound care
Graft Failure [22]	Re-rupture or inadequate healing of the graft, leading to instability.	2% - 10%	Improper graft selection, technical errors, early return to activity	Revision surgery, physical therapy, activity modification
Knee Stiffness [23]	Reduced range of motion due to scar tissue formation or improper healing.	5% - 10%	Delayed rehabilitation, excessive scar formation	Physiotherapy, manipulation under anesthesia, arthroscopic lysis of adhesions
Deep Vein Thrombosis (DVT) [24]	Formation of blood clots in deep veins, typically in the legs, which can lead to pulmonary embolism.	< 1%	Prolonged immobility, smoking, obesity	Anticoagulants, compression therapy, early mobilization
Neurological Complications [25]	Nerve damage results in sensory or motor deficits, commonly affecting the saphenous or peroneal nerve.	< 1%	Surgical technique, anatomical variations	Nerve regeneration therapies, physical therapy, pain management
Osteoarthritis [26]	Degenerative joint disease developing years after surgery is often linked to cartilage damage.	10% - 20% (long-term)	Joint instability, concomitant meniscal injury	Lifestyle modification, joint preservation strategies, pain management

Less common but significant complications

Osteoarthritis Development

Patients who undergo ACL reconstruction surgery are at a significantly higher risk of developing post-traumatic osteoarthritis compared to those who do not have the surgery [27]. Research indicates that approximately 12% of individuals may develop osteoarthritis within five years of the procedure, and around 50% could experience this condition 10 to 17 years after the surgery or initial injury [28]. This long-term risk highlights the importance of ongoing joint health monitoring following ACL reconstruction. The mechanisms linking ACL injury to the subsequent development of osteoarthritis are complex. One primary factor is the initial damage to the articular cartilage at the time of the injury. Additionally, inflammatory processes triggered by the injury and the surgical intervention can contribute to joint degeneration. Altered joint mechanics, loading patterns post-injury, and reduced quadriceps function can further increase the risk of osteoarthritis by compromising the joint's ability to absorb stress effectively. To mitigate the risk of post-traumatic osteoarthritis, several strategies can be employed [3]. Early postoperative rehabilitation is critical for restoring knee motion and quadriceps strength, which helps maintain joint stability. Maintaining a healthy body weight is also essential, as excess weight increases joint loading and stress. Patients should be encouraged to avoid re-injury to the affected knee and engage in regular low-impact exercises to promote joint health and mobility [29].

Patellofemoral Pain Syndrome

Patellofemoral pain syndrome (PFPS) is a notable complication that can arise after ACL reconstruction. This condition is characterized by pain in the front of the knee, often worsened by squatting, stair climbing, or prolonged sitting. The prevalence of PFPS in patients post-ACL surgery can be linked to factors such as altered patellar tracking, muscle imbalances, and joint stiffness [30]. These issues may result from the surgical procedure or activity levels during rehabilitation. Diagnosing PFPS can be challenging because its symptoms overlap with other knee conditions. Accurate diagnosis requires a thorough clinical evaluation, including a detailed history and physical examination. Imaging studies may be used in some cases to exclude other underlying problems. Treatment typically involves a comprehensive approach, including physical therapy to strengthen the quadriceps and improve patellar tracking, modify activities to alleviate pain, and, if necessary, use braces or taping to support the knee during recovery [31].

Hardware-Related Complications

**Figure 1:**
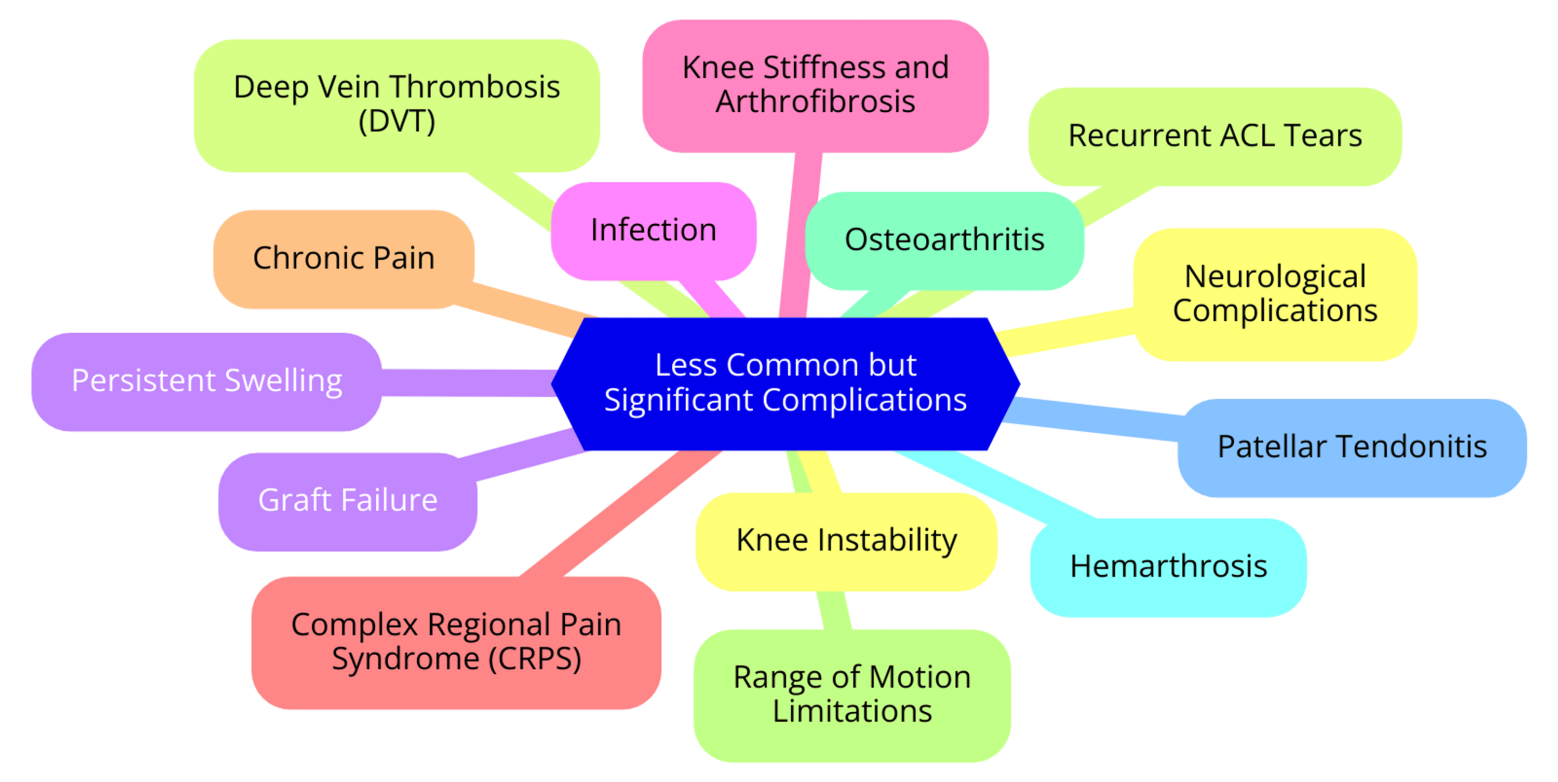
Less common but significant complications Image Credit: Dr Milind R Gharpinde ACL: Anterior Cruciate Ligament

While hardware-related complications following ACL reconstruction are relatively uncommon, they can present significant challenges for patients and healthcare providers. Issues with the fixation devices used to secure the ACL graft may include device rupture or migration, potentially leading to complications such as fractures of the tibia or femur, especially at the bone tunnels created during surgery [19]. Symptoms of hardware-related complications often include persistent pain at the fixation site, swelling, or knee joint instability. Patients may also detect a palpable abnormality over the area where the fixation device is located. Diagnosing these complications can be challenging and often requires advanced imaging techniques, such as CT scans, to visualize the hardware and identify any issues. When hardware complications are detected, surgical intervention is frequently necessary. This typically involves the removal of the malfunctioning hardware, which is usually a straightforward procedure. However, the diagnosis can be complex; in some cases, revision ACL reconstruction may be needed to restore knee function and stability. While uncommon, prompt awareness and management of hardware-related complications are crucial for achieving optimal recovery following ACL surgery [32]. Less common but significant complications are shown in Figure 1.

Factors influencing post-operative complications

Patient-Related Factors

Several patient-related factors significantly influence the risk of postoperative complications following ACL reconstruction. Key considerations include age, gender, and activity level. Younger patients and male athletes often face higher complication rates due to their increased physical demands and activity levels [33]. Pre-existing conditions and comorbidities also play a crucial role. For example, obesity and diabetes mellitus are associated with a greater risk of surgical site infections, which can complicate recovery. Additionally, patient compliance with rehabilitation protocols is vital. Poor adherence to postoperative rehabilitation can lead to complications such as knee stiffness and delayed recovery, highlighting the importance of patient engagement in the recovery process [34].

Surgical Technique and Surgeon Experience

The surgical technique employed during ACL reconstruction and the surgeon's experience influence complication rates. Certain graft types, such as hamstring autografts, have been associated with higher surgical site infections than others. Additionally, including concomitant procedures, like lateral extra-articular tenodesis, can further elevate the risk of complications [35]. The duration of the surgery is also significant; longer operating times have been linked to increased complication rates. Surgeon expertise plays a critical role as well; In contrast, specific data on the correlation between surgeon experience and complication rates may be limited; it is widely recognized that a meticulous surgical technique and thorough familiarity with the procedure can substantially reduce the likelihood of adverse outcomes [36].

Rehabilitation Protocols

Rehabilitation protocols following ACL reconstruction are pivotal in preventing post-operative complications. Early mobilization is crucial for preventing knee stiffness and promoting healing, but it must be managed carefully to avoid overexertion. Physical therapy is central to this process, guiding patients through a structured rehabilitation program emphasizing gradual progression and adherence to safety guidelines. The involvement of a physical therapist is key to ensuring that patients follow a personalized rehabilitation plan, which is essential for minimizing complications. Individualized rehabilitation strategies that address each patient's unique needs, progress, and potential risk factors are critical. Effective communication between the surgical team, physical therapists, and patients is necessary to optimize recovery and improve overall outcomes [37]. Table 2 illustrates factors influencing post-operative complications following ACL tear surgery.

**Table 2:** Factors Influencing Post-Operative Complications Following Anterior Cruciate Ligament (ACL) Tear Surgery DVT: Deep Vein Thrombosis

Factor	Description	Impact on Complications
Patient-Related Factors		
Age [38]	Younger patients often have higher activity levels, while older patients may have comorbidities.	Younger patients may face higher graft failure rates; older patients may experience delayed healing and higher infection risk.
Gender [39]	Differences in anatomical structure, hormonal influences, and muscle strength.	Women may have a higher risk of graft failure and knee stiffness due to anatomical and hormonal differences.
Activity Level [8]	Level of physical activity before and after surgery, including sports participation.	High activity levels increase the risk of graft failure and re-injury.
Comorbidities [40]	Presence of other health conditions such as diabetes, obesity, or cardiovascular disease.	Comorbidities can increase the risk of infection, DVT, and delayed healing.
Compliance with Rehabilitation [41]	Adherence to prescribed physical therapy and rehabilitation protocols.	Poor compliance can lead to complications like knee stiffness, graft failure, and delayed functional recovery.
Surgical Technique and Surgeon Experience		
Graft Selection [42]	Choice of autograft, allograft, or synthetic graft.	Certain graft types may have higher failure rates or cause more postoperative pain.
Technical Precision [2]	Accuracy in graft placement, tensioning, and fixation.	Technical errors can lead to graft failure, instability, and neurological complications.
Surgeon Experience [43]	Experience level of the surgeon performing the ACL reconstruction.	Experienced surgeons typically have lower rates of complications, such as infection and graft failure.
Rehabilitation Protocols		
Early Mobilization [44]	Initiation of movement and weight-bearing activities soon after surgery.	Early mobilization can reduce the risk of knee stiffness and DVT but may increase the risk of graft failure if not properly managed.
Rehabilitation Intensity [44]	The rigor and progression of physical therapy exercises post-surgery.	Overly aggressive rehabilitation may lead to re-injury or graft failure, while inadequate rehab may cause stiffness and poor functional outcomes.
Individualized Rehabilitation [45]	Tailoring the rehabilitation plan to the patient's specific needs and progress.	Personalized rehab can optimize recovery and minimize complications such as muscle atrophy and joint stiffness.

Preventive strategies and best practices

Pre-operative Optimization

Pre-operative optimization is a crucial phase in ensuring a successful ACL reconstruction. One of the most effective strategies is comprehensive patient education and preparation. Patients should be thoroughly informed about the surgical procedure, expected outcomes, and potential complications. This knowledge empowers them to participate in their recovery actively. Emphasizing the importance of adhering to rehabilitation protocols and promptly reporting any concerning symptoms can significantly enhance the likelihood of a smooth recovery [46]. Addressing modifiable risk factors is also vital for optimizing patient health before surgery. Identifying and managing issues such as obesity, smoking, and uncontrolled diabetes can lead to better surgical outcomes. Encouraging patients to adopt healthier lifestyle habits-such as maintaining a balanced diet, engaging in regular physical activity, and quitting smoking, can reduce the risk of complications and improve overall recovery [47].

Intraoperative Techniques

During the surgical procedure, techniques that minimize tissue trauma and reduce the risk of infection are crucial. Surgeons should adhere to strict sterile techniques and maintain a clean operating room environment. The administration of prophylactic antibiotics before surgery is also essential for preventing postoperative infections, which, while rare, can have serious consequences for recovery. Another critical consideration is the selection of graft type and fixation method. Surgeons must carefully evaluate graft strength, size, and the fixation technique to ensure optimal outcomes. Choosing the appropriate graft and fixation method can significantly influence the risk of graft-related complications, such as re-tear or failure [21].

Post-operative Care

Post-operative care is as crucial as pre-operative preparation and intraoperative techniques. Close monitoring for early signs of complications- such as infection, hemarthrosis, and DVT-is essential for prompt intervention. Patients should be educated about symptoms to watch for and encouraged to report any concerns immediately, as early detection can improve the management of potential issues. The importance of follow-up appointments cannot be overstated. Regular surgical and rehabilitation team visits are critical for tracking progress and addressing potential complications. Adherence to rehabilitation protocols is vital for achieving optimal outcomes, and ongoing communication with healthcare providers helps reinforce the importance of this commitment [48]. Effective pain management is also essential for ensuring patient comfort and facilitating adherence to rehabilitation. A multimodal approach that combines various pain relief methods, such as nonsteroidal anti-inflammatory drugs (NSAIDs), acetaminophen, and nerve blocks, can help minimize opioid use and improve overall recovery outcomes. By implementing these preventive strategies and best practices, healthcare providers can significantly reduce the risk of complications following ACL reconstruction and enhance the overall patient experience. Table 3 outlines preventive strategies and best practices for minimizing post-operative complications following ACL tear surgery [49].

**Table 3:** Preventive Strategies and Best Practices for Minimizing Post-Operative Complications Following Anterior Cruciate Ligament (ACL) Tear Surgery DVT: Deep Vein Thrombosis

Preventive Strategy/Best Practice	Description	Targeted Complications	Outcome
Preoperative Assessment and Optimization [50]	Comprehensive evaluation of the patient’s health status and risk factors before surgery.	Infection, DVT, delayed healing	Reduces the risk of complications by addressing modifiable factors (e.g., smoking cessation, glycemic control).
Patient Education and Counseling [51]	Educating patients about the importance of adherence to rehabilitation and activity modifications post-surgery.	Graft failure, re-injury, poor functional recovery	Enhances patient compliance with rehabilitation protocols, reducing the risk of graft failure and knee stiffness.
Surgical Technique Precision [13]	Ensuring accurate graft placement, tensioning, and fixation using advanced techniques and technologies.	Graft failure, knee instability, neurological complications	Improves graft stability and reduces technical errors leading to complications.
Choice of Graft Type [52]	Selecting the most appropriate graft type based on the patient’s age, activity level, and preference.	Graft failure, postoperative pain	Customizes graft selection to reduce failure rates and improve patient satisfaction.
Infection Control Measures [21]	Implementing strict aseptic techniques, prophylactic antibiotics, and sterile surgical environments.	Infection	Minimizes the risk of postoperative infections, enhancing recovery and outcomes.
Thromboprophylaxis [53]	Anticoagulants, compression stockings, and early mobilization are used to prevent blood clots.	DVT, pulmonary embolism	Reduces the incidence of DVT and related complications, ensuring safer recovery.
Early and Structured Rehabilitation [20]	Initiating early but controlled physical therapy focusing on range of motion and strength.	Knee stiffness, muscle atrophy	Promotes quicker recovery of knee function while minimizing the risk of stiffness and atrophy.
Individualized Rehabilitation Protocols [20]	Tailoring the rehabilitation process to the patient’s progress, injury severity, and personal goals.	Re-injury, poor functional outcomes	Optimizes recovery by adjusting rehabilitation intensity to patient needs, preventing over- or under-loading.
Regular Follow-Up and Monitoring [54]	Conducting regular post-operative visits to monitor progress and identify complications early.	All complications (infection, graft failure, stiffness, etc.)	Early detection and intervention of complications, improving long-term outcomes.
Advanced Pain Management [55]	Utilizing multimodal pain management strategies to control postoperative pain and facilitate rehabilitation.	Chronic pain, poor compliance with rehabilitation	Enhances patient comfort, enabling better participation in rehabilitation and faster recovery.

Future directions in ACL surgery and complication management

Future directions in ACL surgery and complication management are shaped by advancements in surgical techniques, evolving rehabilitation practices, and ongoing research into complication prevention. Innovations in surgical techniques include the development of biologically engineered ACL grafts that replicate the properties of the native ligament. These grafts aim to enhance mechanical characteristics and promote better tissue integration, potentially reducing complications associated with traditional autologous and allogenic grafts [5]. Improved arthroscopic techniques have also made ACL reconstruction less invasive and more precise. For example, independent tunnel drilling allows for more accurate replication of the anatomic femoral origin of the ACL, which may improve outcomes and reduce complications. Additionally, finite element modeling is an emerging technology that simulates complex knee joint biomechanics, facilitating individualized surgical planning and optimizing ACL reconstruction strategies tailored to each patient [5]. Rehabilitation protocols are increasingly transitioning from fixed timelines to criterion-based progression. This approach emphasizes achieving specific functional milestones before advancing to the next phase of rehabilitation, which can enhance recovery outcomes and reduce the risk of complications [56]. New trends in rehabilitation focus on multimodal pain management and accelerated protocols that minimize opioid use and promote early mobilization. These strategies aim to improve patient satisfaction and expedite recovery while ensuring safety. Integrating telehealth in rehabilitation further supports better patient monitoring and adherence to rehabilitation protocols, allowing for timely interventions and adjustments based on patient progress [56]. Ongoing research explores biomechanical factors contributing to ACL injuries and complications post-surgery. Studies are investigating the effects of different graft types and surgical techniques on knee stability and function. Efforts to prevent complications such as infection, DVT, and graft failure include optimizing surgical protocols, enhancing postoperative care, and developing better monitoring systems to detect complications early. Long-term studies are necessary to assess the outcomes of new surgical techniques and rehabilitation protocols, helping to establish best practices and refine approaches to minimize complications associated with ACL surgery [19].

## Conclusions

In conclusion, ACL reconstruction surgery remains a critical intervention for restoring knee stability and enabling individuals to return to their pre-injury activities; it is not challenging. The potential for postoperative complications, ranging from infections and graft failures to long-term issues like osteoarthritis and neurological impairments, underscores the need for careful patient selection, meticulous surgical technique, and comprehensive post-operative care. Understanding the various factors contributing to these complications, including patient characteristics, surgical methods, and rehabilitation protocols, is essential for optimizing outcomes. By addressing these challenges through preventive strategies and evidence-based practices, healthcare providers can enhance the overall success of ACL reconstruction, ultimately improving the quality of life for patients and reducing the burden of long-term disability. This review highlights the importance of a multidisciplinary approach in managing post-operative complications. It emphasizes the need for ongoing research and innovation to refine surgical techniques and rehabilitation strategies, ensuring better outcomes for all patients undergoing ACL surgery.
